# Diagnosis of extraskeletal myxoid chondrosarcoma in the thigh using *EWSR1*-*NR4A3* gene fusion: a case report

**DOI:** 10.1186/s13256-016-1113-2

**Published:** 2016-11-10

**Authors:** Hiroki Kobayashi, Kazutaka Kikuta, Tetsuya Sekita, Michiro Susa, Kazumasa Nishimoto, Aya Sasaki, Kaori Kameyama, Shintaro Sugita, Tadashi Hasegawa, Masaya Nakamura, Morio Matsumoto, Hideo Morioka

**Affiliations:** 1 Department of Orthopaedic Surgery, Keio University School of Medicine, 35 Shinanomachi, Shinjuku-ku, 160-8582 Tokyo Japan; 2 Department of Pathology, Keio University School of Medicine, Tokyo, Japan; 3 Department of Surgical Pathology, Sapporo Medical University School of Medicine, Sapporo, Japan

**Keywords:** Extraskeletal myxoid chondrosarcoma, Fluorescence *in situ* hybridization, *EWSR1-NR4A3*

## Abstract

**Background:**

Extraskeletal myxoid chondrosarcoma is a rare soft tissue sarcoma that has unusual ultrastructural and molecular features. However, unlike other soft tissue sarcomas, it does not have specific clinical symptoms or radiological features, which can make its diagnosis difficult. Nevertheless, extraskeletal myxoid chondrosarcoma has a rare gene fusion (*EWSR1-NR4A3*) that is useful for making a differential diagnosis.

**Case presentation:**

A 43-year-old Japanese man presented with a soft tissue mass in his right thigh. A physical examination and radiography revealed a large soft tissue mass. During magnetic resonance imaging, the mass exhibited isointensity on T1-weighted images and high intensity on T2-weighted images, as well as gadolinium enhancement at the side edge of the partition structure. Thus, we considered a possible diagnosis of a malignant myxoid soft tissue tumor, such as myxoid liposarcoma, myxofibrosarcoma, or metastatic carcinomas, including myoepithelial tumor and neuroendocrine tumor, and performed an incisional biopsy to make a definitive diagnosis. The pathological findings revealed a lobulated tumor with a myxoid structure and atypical spindle-shaped cells that created eosinophilic cord-like forms. Immunohistochemistry revealed that the tumor was positive for S-100 and negative for synaptophysin, chromogranin A, and pan keratin (AE1/AE3). The percentage of Ki-67 was 10 % in the hot spot area. Based on these clinicopathological findings, we initially considered the possibility of a myxoid liposarcoma, although we did not observe any lipoblasts. Therefore, we considered the possibility of an extraskeletal myxoid chondrosarcoma. As this tumor is very rare, we searched for the *EWSR1-NR4A3* gene fusion using fluorescence *in situ* hybridization, which confirmed the diagnosis of extraskeletal myxoid chondrosarcoma. Positron emission tomography-computed tomography did not identify any obvious metastases, and we performed radical resection of our patient’s vastus medialis and femur with a 3 cm margin. After the resection, we treated his resected femur using liquid nitrogen, and reconstructed his femur using autogenous fibula and plate fixation. No local recurrence or metastasis was observed at the 1-year follow-up.

**Conclusion:**

Genetic testing is useful for diagnosing extraskeletal myxoid chondrosarcoma based on the presence of the *EWSR1-NR4A3* gene fusion.

## Background

Extraskeletal myxoid chondrosarcoma (EMC) accounts for 2.5 to 3 % of all soft tissue sarcomas, and is characterized by a multinodular architecture, myxoid matrix, and malignant chondroblasts [1]. Oliveira *et al*. defined EMC as a distinct sarcoma with a chondroblastic origin that arises from the extraskeletal soft tissues [2]. However, the histogenesis of EMC remains controversial, and EMC is currently classified as a tumor of uncertain differentiation in the revised version of the *WHO Classification of Tumours of Soft Tissue and Bone* [3]. Furthermore, EMC does not have specific clinical, imaging, or pathological characteristics, which makes it difficult to definitively differentiate between EMC and other myxoid tumors. However, EMC has a rare gene fusion, EWS RNA binding protein 1-nuclear receptor subfamily 4, group A, member 3 (*EWSR1-NR4A3*), which is useful in making a differential diagnosis [4, 5]. Therefore, we report the case of a 43-year-old man with an EMC in his right thigh, which we definitively diagnosed based on the presence of the *EWSR1-NR4A3* gene fusion.

## Case presentation

**Fig. 1:**
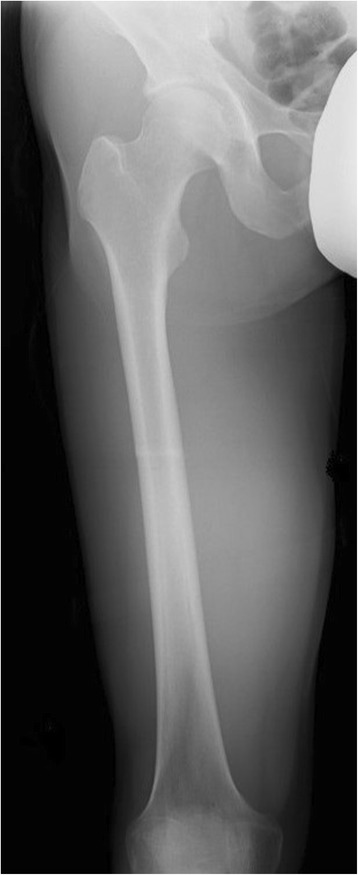
Radiography reveals a soft tissue mass with no calcification or periosteal reaction

**Fig. 2:**
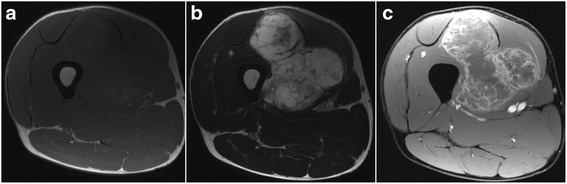
Magnetic resonance imaging findings. **a** T1-weighted axial imaging reveals a soft tissue tumor with an isointense signal. **b** T2-weighted axial imaging reveals a soft tissue tumor with a high-intensity signal. **c** Axial imaging with gadolinium enhancement at the side edge of the partition structure

**Fig. 3:**
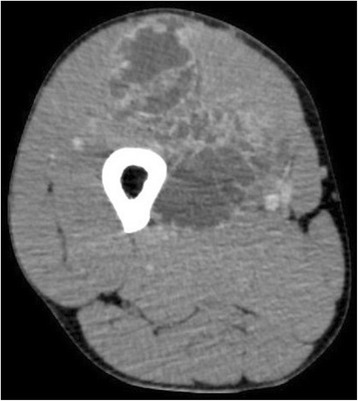
Computed tomography reveals a tumor without calcification or femur infiltration

**Fig. 4:**
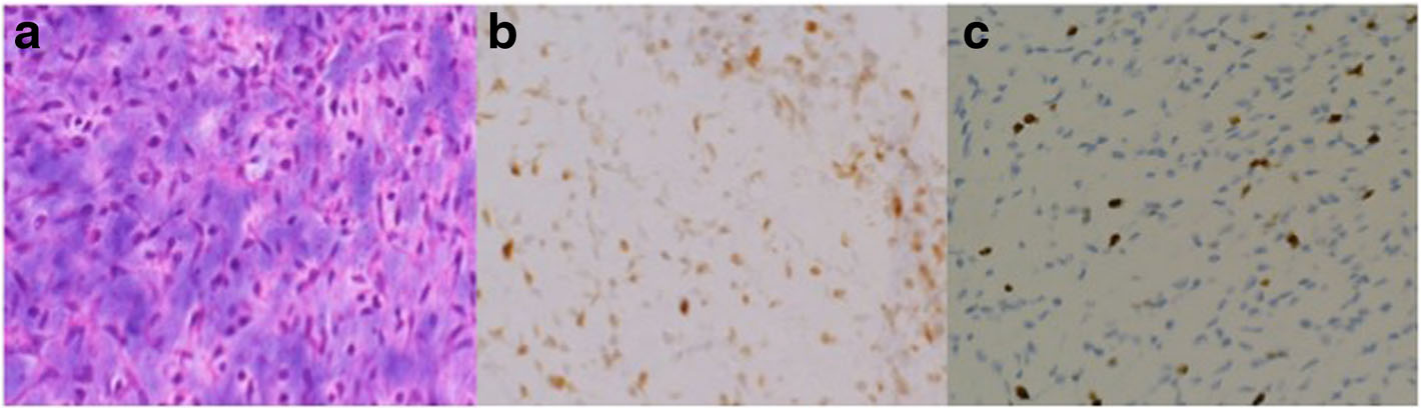
Pathological and immunohistochemical findings. **a** Hematoxylin and eosin staining (×200). **b** Immunohistochemistry reveals positive expression of S-100. **c** Immunohistochemistry reveals that the percentage of Ki-67 was 10 % (×200)

**Fig. 5:**
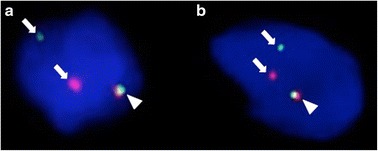
Fluorescence *in situ* hybridization with two split signals (*EWSR1* and *NR4A3*). Both of *EWSR1* and *NR4A3* split signals showed a pair of split (*arrow*) and fused (*arrow head*) patterns of red and green dyes. We counted 50 nuclei of tumor cells and they were assessed as positive if more than 10 % of tumor cells showed split signals. **a** With *EWSR1*, the split signal was detected in 44/50 (88 %) of the tumor cells. **b** With *NR4A3*, the split signal was detected in 47/50 (94 %) of the tumor cells

**Fig. 6:**
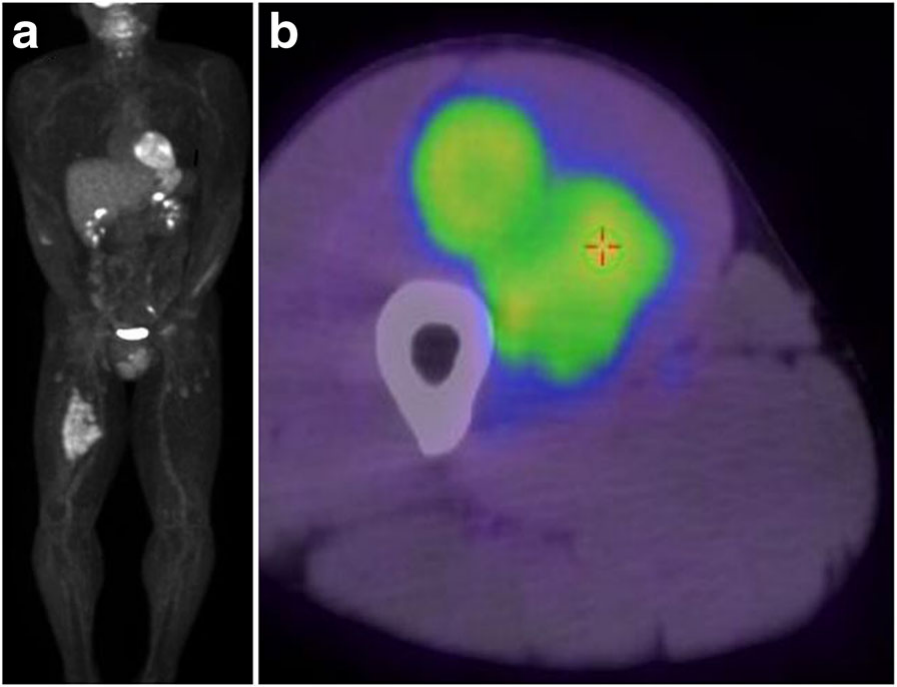
**a** Positron emission tomography-computed tomography reveals no obvious distant metastases. **b** An axial image with the maximum standardized uptake value of 3.1 (at the crosshairs)

**Fig. 7:**
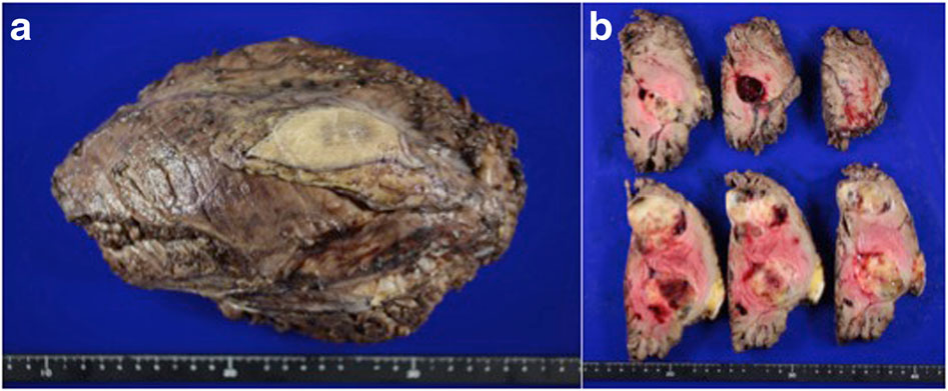
**a** Gross appearance of the resected tumor. **b** Macroscopic findings of coronal section of resected tumor. Cystic cavities, hemorrhage and necrosis are found in the tumor. The tumor has a well-defined lobular architecture defined by fibrous septa

**Fig. 8:**
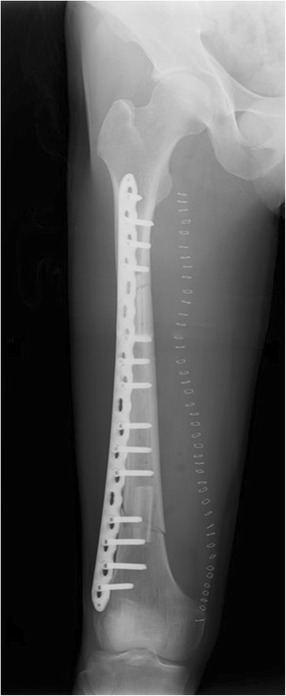
Postoperative radiography after the femur reconstruction

A 43-year-old Japanese man presented with a soft tissue mass in his right thigh during June 2015, which he originally noticed in January 2015. He did not have any relevant personal or family history, and a physical examination revealed a hard and elastic mass (approximate size 30 cm) that did not exhibit redness, inflammation, or a pulse. His blood test results were normal, and radiography revealed a large soft tissue mass without calcification, periosteal reaction, or infiltration into his femur (Fig. 1). Magnetic resonance imaging (MRI) revealed a mass with an isointense signal on T1-weighted images and a high-intensity signal on T2-weighted images, with gadolinium enhancement on the side edge of the partition structure (Fig. 2). Computed tomography (CT) also revealed no tumor calcification or obvious infiltration into his femur (Fig. 3). Based on these findings, we considered a possible diagnosis of a malignant myxoid soft tissue tumor, such as myxoid liposarcoma, myxofibrosarcoma, or metastatic carcinomas, including myoepithelial tumor and neuroendocrine tumor, and performed an incisional biopsy to make a definitive diagnosis. The pathological findings revealed a lobulated tumor with a myxoid matrix and atypical spindle-shaped cells that created an eosinophilic cord-like structure (Fig. 4a). We also observed that the tumor was positive for S-100 (Fig. 4b) which is mainly present in neurons, chondrocytes, adipocytes, and pigment cells, and negative for synaptophysin, chromogranin A, and pan keratin (AE1/AE3). The percentage of Ki-67 was 10 % in the hot spot area (Fig. 4c). Thus, based on the histology results, the site of occurrence, and our patient’s age, we considered a differential diagnosis of myxoid liposarcoma, which is composed of uniform round-to-oval cells and lipoblasts in a prominent myxoid matrix with delicate arborizing vasculature. However, the tumor in the present case did not contain the characteristic lipoblasts and capillary vasculature. Thus, we also considered the possibility of an EMC, despite its rare nature, as this diagnosis agreed with our patient’s age, site of occurrence, and myxoid pathology. Therefore, we performed fluorescence *in situ* hybridization (FISH) to differentiate between EMC and myxoid liposarcoma. This evaluation identified the *EWSR1-NR4A3* gene fusion, which convinced us that he had an EMC (Fig. 5). We also performed systemic positron emission tomography-CT, which did not identify any obvious metastases (Fig. 6). The tumor was classified as TNM stage IIb, and we performed extensive resection of his tumor (Fig. 7) and femur with a 3 cm margin, as the tumor was in contact with his femur. His femur was treated using liquid nitrogen, and reconstructed using autogenous fibula and internal plate fixation (Fig. 8). Our patient was free from recurrence and metastases at the 1-year follow-up.

## Discussion

In the present case, we observed an EMC that arose in the deep soft tissues of our patient’s right thigh. The lower extremities are a typical location for this lesion, and other reports have also described EMCs occurring in the finger, hip joint, head, neck, chest wall, and abdominal wall [6, 7]. Furthermore, previous reports have indicated that EMCs are typically indolent and associated with long-term survival (even in cases of metastatic disease), although aggressive clinical courses are occasionally observed. The risk factors for a poor clinical outcome are a large tumor size (>10 cm), anaplasia, and high mitotic activity [8]. The tumor in the present case had a diameter of approximately 30 cm, which suggests that it was probably an aggressive tumor. Unfortunately, the roles of chemotherapy and radiotherapy for EMC are poorly defined; ifosfamide-based regimens or doxorubicin-based regimens are not considered effective, although radiotherapy may be beneficial in the adjuvant setting or as palliative treatment for metastatic disease. Thus, we performed extensive resection of the vastus medialis and femur, which resulted in our patient being recurrence-free at the 1-year follow-up, although close long-term follow-up is necessary to confirm that this treatment provided long-term efficacy. Therefore, an early accurate diagnosis and complete resection are important for curing cases of EMC [9].

The difficulty in definitively diagnosing EMC is related to differentiating between EMC and other myxoid soft tissue sarcomas, such as myxofibrosarcoma, myxoid liposarcoma, and metastatic carcinomas, including myoepithelial tumor and neuroendocrine tumor, as EMCs do not have specific clinical characteristics. In most cases, the tumor arises as a single slow-growing soft tissue lesion that has a diameter of approximately 5 to 10 cm [10]. During CT, EMCs appear as soft tissue masses with lobular contours, although there are no characteristic radiological features that can differentiate EMC from other malignancies [10]. The present case highlights these nonspecific features, as the tumor gradually grew to a diameter of 30 cm in his right thigh, although CT did not reveal any obvious femur infiltration and there was no qualitative evaluation of the tumor. During MRI, it is best to use settings that can reveal the myxoid stroma of the EMC, although EMCs do not have any distinct MRI features because the characteristic features of most myxoid tumors are soft tissue masses with high-intensity signals on T2-weighted images [6]. The cytological features of EMC are round-to-oval cells that form cords in an abundant and brightly metachromatic chondromyxoid background [7]. Although EMC is considered a chondrosarcoma, it does not typically exhibit a cartilage matrix, which also makes it difficult to differentiate between EMC and other myxoid tumors. Therefore, MRI and histopathology alone are probably insufficient to distinguish EMC from other myxoid soft tissue tumors.

Previous reports have indicated that there is an EMC-specific gene fusion, as approximately two-thirds of EMCs harbor the chromosomal reciprocal translocation of t(9;22)(q22;q12) [11], which is the fusion of the *EWSR1* gene to the *NR4A3* gene [4, 5]. Furthermore, the *EWSR1*/*NR4A3* gene fusion is the most common genetic event in EMC, as it occurs in 62 % of cases with a classical morphology [12]. In this context, FISH is considered the most useful genetic test for non-skeletal lesions, based on their heterogeneous cell populations and karyotypic variability [13]. The present case also confirms the usefulness of FISH for non-skeletal lesions, as we were able to make a definitive diagnosis of EMC based on the detection of the *EWSR1-NR4A3* gene fusion. Therefore, it is probably appropriate to perform genetic testing for the *EWSR1-NR4A3* gene fusion in cases of suspected EMC.

## Conclusions

In the present case, we suspected EMC because of findings from various examinations, and used genetic testing to definitively diagnose EMC in our patient’s right thigh based on the presence of the *EWSR1-NR4A3* gene fusion. Our patient has remained recurrence-free for 1-year after extensive resection, although long-term follow-up is necessary to confirm the efficacy of this treatment. Therefore, genetic testing should be considered in similar cases, as the definitive diagnosis of EMC is complicated by the absence of specific clinical characteristics.

## Abbreviations

CT: Computed tomography
EMC: Extraskeletal myxoid chondrosarcoma
*EWSR1*: EWS RNA binding protein 1
FISH: Fluorescence *in situ* hybridization
MRI: Magnetic resonance imaging
*NR4A3*: Nuclear receptor subfamily 4, group A, member 3
